# Understanding the Radioactive Ingrowth and Decay of Naturally Occurring Radioactive Materials in the Environment: An Analysis of Produced Fluids from the Marcellus Shale

**DOI:** 10.1289/ehp.1408855

**Published:** 2015-04-02

**Authors:** Andrew W. Nelson, Eric S. Eitrheim, Andrew W. Knight, Dustin May, Marinea A. Mehrhoff, Robert Shannon, Robert Litman, William C. Burnett, Tori Z. Forbes, Michael K. Schultz

**Affiliations:** 1 Interdisciplinary Human Toxicology Program, University of Iowa, Iowa City, Iowa, USA; 2 University of Iowa State Hygienic Laboratory, Research Park, Coralville, Iowa, USA; 3 Department of Chemistry, University of Iowa, Iowa City, Iowa, USA; 4 Quality Radioanalytical Support, Grand Marais, Minnesota, USA; 5 Radiochemistry Laboratory Basics, The Villages, Florida, USA; 6 Department of Earth, Ocean and Atmospheric Science, Florida State University, Tallahassee, Florida, USA; 7 Department of Radiology, and; 8 Department of Radiation Oncology, Free Radical and Radiation Biology Program, University of Iowa, Iowa City, Iowa, USA

## Abstract

**Background:**

The economic value of unconventional natural gas resources has stimulated rapid globalization of horizontal drilling and hydraulic fracturing. However, natural radioactivity found in the large volumes of “produced fluids” generated by these technologies is emerging as an international environmental health concern. Current assessments of the radioactivity concentration in liquid wastes focus on a single element—radium. However, the use of radium alone to predict radioactivity concentrations can greatly underestimate total levels.

**Objective:**

We investigated the contribution to radioactivity concentrations from naturally occurring radioactive materials (NORM), including uranium, thorium, actinium, radium, lead, bismuth, and polonium isotopes, to the total radioactivity of hydraulic fracturing wastes.

**Methods:**

For this study we used established methods and developed new methods designed to quantitate NORM of public health concern that may be enriched in complex brines from hydraulic fracturing wastes. Specifically, we examined the use of high-purity germanium gamma spectrometry and isotope dilution alpha spectrometry to quantitate NORM.

**Results:**

We observed that radium decay products were initially absent from produced fluids due to differences in solubility. However, in systems closed to the release of gaseous radon, our model predicted that decay products will begin to ingrow immediately and (under these closed-system conditions) can contribute to an increase in the total radioactivity for more than 100 years.

**Conclusions:**

Accurate predictions of radioactivity concentrations are critical for estimating doses to potentially exposed individuals and the surrounding environment. These predictions must include an understanding of the geochemistry, decay properties, and ingrowth kinetics of radium and its decay product radionuclides.

**Citation:**

Nelson AW, Eitrheim ES, Knight AW, May D, Mehrhoff MA, Shannon R, Litman R, Burnett WC, Forbes TZ, Schultz MK. 2015. Understanding the radioactive ingrowth and decay of naturally occurring radioactive materials in the environment: an analysis of produced fluids from the Marcellus Shale. Environ Health Perspect 123:689–696; http://dx.doi.org/10.1289/ehp.1408855

## Introduction

New unconventional drilling technologies (horizontal drilling combined with hydraulic fracturing, called “fracking”) are unlocking vast reserves of natural gas in the United States and around the world (Cueto-Felgueroso and Juanes 2013; U.S. Energy Information Administration 2014). The potential economic value of these reserves has stimulated a rapid globalization of the approach (Boyer et al. 2011). However, the pace of proliferation of these practices has raised concerns about the potential for unintended and undesirable environmental impacts (Finkel 2011; Goldstein et al. 2012; Howarth et al. 2011; Kerr 2010; Schmidt 2011; Thompson 2012). One key environmental issue associated with unconventional drilling and hydraulic fracturing is the management of water resources and liquid wastes (flowback and produced fluids) (Clark and Veil 2009; Kondash et al. 2014; Lutz et al. 2013; Vidic et al. 2013; Yang et al. 2013; Zhang et al. 2014). Of the environmental contaminants documented in hydraulic fracturing liquid wastes, naturally occurring radioactive materials (NORM) are of particular concern (Brown 2014; Kargbo et al. 2010; Vengosh et al. 2014).

Recent attention has focused on unintentional releases of radium (Ra) isotopes from wastewater treatment plants (Warner et al. 2013), which can arise from incomplete treatment of high ionic strength flowback and produced fluids (Gregory et al. 2011). For example, breakthrough of untreated fluids at a waste treatment facility in central Pennsylvania (northeastern United States) led to Ra contamination in stream sediments measured to be a factor of 200 greater in radioactivity concentration than local background levels (Warner et al. 2013). The magnitude of the Ra contamination at this site prompted the plant operator to proceed with remediation of contaminated sediments in the surface water system (Blacklick Creek) impacted by the discharges (Hunt 2014). Thus, NORM contamination of local environments, arising from improper treatment and disposal of produced fluids, could emerge as an unintended consequence of hydraulic fracturing. Although the potential for local populations and workers to experience unhealthy exposures to NORM contained in such wastes is controversial (Brown 2014), monitoring the radioactivity concentrations in these materials is critical to the development of effective waste management strategies and exposure assessments. However, few peer-reviewed reports are available that document levels of NORM in produced fluids. Of those available from the Marcellus Shale (the largest shale-gas formation in the United States), most report radioactivity concentrations of a single element—Ra (Barbot et al. 2013; Haluszczak et al. 2013; Nelson et al. 2014; Rowan et al. 2011).

**Figure 1:**
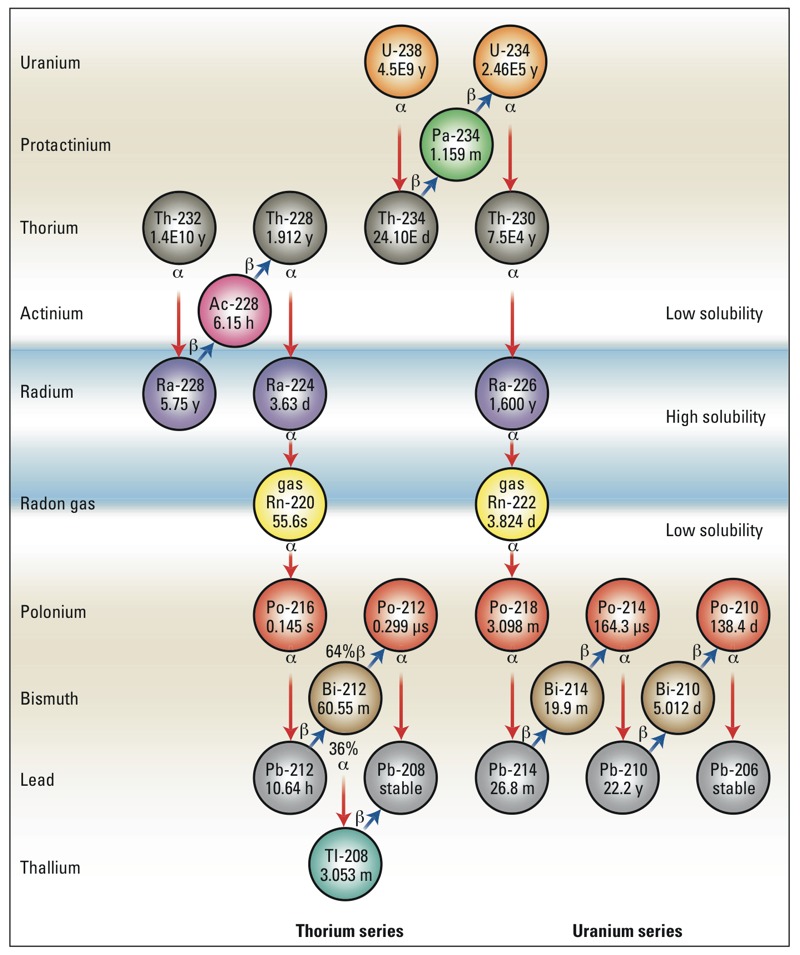
Natural thorium and uranium decay chains. Half-lives and decay information were obtained from the NuDat 2 Database (NNDC 2013). Abbreviations: d, days; h, hours; m, minutes; s, seconds; y, years.

The naturally occurring Ra isotopes of concern (^226^Ra and ^228^Ra) have been reported (in peer-reviewed literature) to exceed 670 Bq/L and 95 Bq/L, respectively, in produced fluids (Barbot et al. 2013; Haluszczak et al. 2013; Nelson et al. 2014; Rowan et al. 2011). However, little attention has been paid to other environmentally persistent alpha- and beta-emitting NORM such as uranium (U), thorium (Th), radon (Rn), bismuth (Bi), lead (Pb), and polonium (Po) isotopes (Figure 1). In reviewing a report of gross alpha levels in fluids from Marcellus Shale, we observed that reported Ra radioactivity concentrations were similar to maximum gross alpha levels (Barbot et al. 2013), indicating that Ra had been selectively extracted into the liquid wastes, while alpha-emitting daughters remained insoluble under the geochemical conditions of the fluid extraction process. Given that Ra decay products had likely existed in a steady-state radioactive equilibrium with Ra isotopes in the solid shale-formation matrix for millions of years prior to drilling activities, these observations prompted us to explore the radioactive equilibrium relationships of Ra decay products in produced fluids, particularly for the longer-lived alpha-emitters, ^228^Th (*t*_1/2_ = 1.91 years) and ^210^Po (*t*_1/2_ = 138 days) (half-lives were extracted from the NuDat 2 Database) [National Nuclear Data Center (NNDC) 2013].

As we reported previously, the chemical composition of fluids from the Marcellus Shale can interfere with the analysis of Ra isotopes by wet chemistry methods (Nelson et al. 2014). However, the physicochemical properties of select alpha-emitters (^210^Po, ^228^Th, and certain U isotopes) allow for chemical extraction and analysis by isotope dilution alpha spectrometry techniques. Thus, we developed a method to analyze alpha-emitting Po, Th, and U isotopes in produced fluids from the Marcellus Shale. Using this method (in systems closed to the release of gaseous radon), we found that estimates of total radioactivity in produced fluids based on Ra isotopes alone can underestimate the total radioactivity present due to the ingrowth of Ra decay-product radionuclides, a process that we demonstrate can be modeled using radioactive ingrowth equations (Bateman 1910). This model predicts that when produced fluids are sealed to the release of radon gas, the total radioactivity concentration of produced fluid can increase by a factor greater than five within the first 15 days following extraction due to the ingrowth of Ra decay products. Measurements of decay series radionuclides ^210^Po and ^228^Th in produced fluids from the Marcellus Shale presented here support these predictions. Thus, estimates of the radioactivity associated with hydraulic fracturing liquid wastes must include projections of ingrowth of decay product radionuclides in the natural uranium (^238^U) and thorium (^232^Th) decay series.

## Methods

*General*. The State Hygienic Laboratory (SHL) at the University of Iowa is accredited by the U.S. National Environmental Laboratory Accreditation Program (NELAP). Standard operating procedures and quality assurance measures meet those established by NELAP. All chemical reagents used were ACS grade or higher. All radioactivity values were decay corrected to the reference date of 7 May 2013, 0800 hours (CST). All uncertainties, unless indicated, are standard uncertainties corresponding to one standard deviation of multiple measurements (Currie 1968).

*Tracers and standards*. All radioactive tracers were *a*) standard reference materials (SRMs) obtained from the U.S. National Institute of Standards and Technology (NIST), *b*) NIST-traceable certified reference materials (CRMs) obtained from Eckert & Ziegler Radioisotopes (E&Z) or Analytics, or *c*) SRMs obtained from the United Kingdom National Physical Laboratory (NPL) Management Ltd. The following sources were used: a 3-L liquid Marinelli geometry (E&Z 93474), ^210^Pb (E&Z 94643), ^nat^U (E&Z CRM 92564), ^232^U (E&Z CRM 92403 or E&Z 7432; certified in equilibrium with ^228^Th), ^230^Th (NIST SRM 4342A or Analytics 67900-294), ^209^Po (NIST 4326 or E&Z CRM 92565), and multiline alpha-emitting sources (E&Z 91005, Analytics 59956-121, and Amersham AMR.43).

*Sample description*. A representative sample of produced fluids from northeastern Pennsylvania (Nelson et al. 2014) was used for all of the following experiments. A 200-L drum of Marcellus Shale produced fluids was received at the SHL on 7 May 2013. The sample originated from a well that was horizontally drilled to a depth of 2,100 m and fractured with approximately 35,000 m^3^ of hydraulic fracturing fluid in early 2012. Analysts at SHL characterized the elemental composition using standard techniques.

*High purity germanium (HPGe) gamma spectrometry*. HPGe gamma spectrometry of produced fluids was conducted as previously described (Nelson et al. 2014). Briefly, we calibrated our detector to a 3-L liquid Marinelli geometry (E&Z 93474) using standard practices. To calibrate for the low energy gamma emission of ^210^Pb, we counted a 3-L Marinelli beaker spiked with ^210^Pb of known activity (E&Z 94643). This spectrum was merged with the detector calibration using standard features available in ORTEC Gamma Vision (version 6.08, analysis engine Env32). Quality assurance and quality control (QA/QC) measures included weekly background counts, and linearity and efficiency checks collected three times per week. A 3-L sample was homogenized by heating with 51 g of Bacto Agar (BD 214010; Becton Dickinson) and allowed to cool in a 3-L Marinelli beaker. The sample was then counted for 17 hr on a 30% efficient ORTEC HPGe. Spectral analysis was performed using ORTEC Gamma Vision (Version 6.08) with a library of radionuclides created in GammaVision Library Editor according to the manufacturer’s recommendations. All emission energies, half-lives (except for that of ^209^Po), and their uncertainties were extracted from the NuDat 2 Database (NNDC 2013) and include evaluated nuclear data at the time of analysis. The sole exception was the half-life of ^209^Po, for which we chose to use 128.3 years (Collé et al. 2007).

*Alpha-emitting radionuclides*. Analysis of produced fluids for alpha-particle emitting radionuclides in the ^238^U and ^232^Th decay series (^210^Po, ^228^Th, ^230^Th, ^234^U, ^235^U, ^238^U) was conducted by preconcentration and isotope dilution alpha spectrometry. All results presented are from an unfiltered subsample (20 L, in a polypropylene carboy) drawn from the homogenized 200-L barrel. Following each subsampling, the barrel was hermetically sealed. The subsample pH was adjusted to 2 and held (approximately 48 hr) to allow iron-rich particulate to dissolve to a transparent, yellowish acidified solution. Preconcentration and matrix simplification were then conducted via coprecipitation of Po, Th, and U with endogenous iron (Fe) as the hydroxide [Fe(OH)_3_] and added manganese (Mn) for coprecipitation as manganese dioxide (MnO_2_), as previously described (Eichrom Technologies, LLC 2009; Harada et al. 1989). Preliminary experiments demonstrated exceedingly low concentrations of ^230^Th, allowing use of ^230^Th as a radiotracer to determine yields and concentration of ^228^Th. Following preconcentration and matrix simplification [via metal oxide/hydroxide co-precipitation; i.e., Fe(OH)_3_ and MnO_2_], Po, U, and Th were separated into radiochemically pure fractions via extraction chromatography.

*MnO_2_ coprecipitations*. Samples were spiked with 150–500 mBq of ^209^Po, ^230^Th, ^232^U, and ^nat^U. After appropriate tracers were added, MnO_2_ coprecipitations were performed, based on published methods (Burnett et al. 2012; Moore 1976; Nour et al. 2004). Potassium permanganate (15 or 30 mg) and bromocresol purple (1 mL, 0.1%) were added to acidified (pH < 2) produced fluid (0.5 L) in glass beakers. The sample was diluted 2-fold in distilled water (dH_2_O), covered with a watch glass, and boiled (1 hr). The pH was adjusted to 7–8, and the sample was boiled for 1 hr and cooled overnight. Following the cooling period, the supernatant was aspirated; the remaining slurry (~ 50 mL) was transferred to a plastic conical tube (50 mL) and centrifuged (10 min), and the supernatant was discarded. Beakers were washed twice (5 mL 6 M HCl; 1 mL 1 M ascorbic acid), each time transferring the wash liquid to the 50-mL centrifuge tube to dissolve the MnO_2_ pellets. Centrifuge tubes were then gently heated in a water bath to fully dissolve the pellet and clarify the solution.

*Method 1: SR resin and silver (Ag) autodeposition separation of polonium*. In some cases, Po isotopes were isolated following an Eichrom method (Eichrom Technologies LLC 2009). Briefly, samples were spiked with ^209^Po prior to MnO_2_ or Fe(OH)_3_ precipitation. Precipitates were dissolved (10 mL, 2 M HCl), reduced (1 mL, 1 M ascorbic acid), and gently heated in a water bath. Solutions were then loaded onto preconditioned Eichrom SR Resin (10 mL, 2 M HCl). Columns were rinsed (10 mL, 2 M HCl) to remove trace contaminants. Po was then eluted with two additions of acid [5 mL, 1 M nitric acid (HNO_3_); 15 mL, 0.1 M HNO_3_]. Eluent was wet ashed (0.5 M HCl) under low heat to remove HNO_3_. Samples were then dissolved (40 mL, 0.5 M HCl) and reduced (100 mg ascorbic acid). Po was then allowed to autodeposit overnight at 80°C onto Ag disks painted on one side with acid-resistant acrylic paint. Disks were then cleaned (~ 10 mL 0.5 M HCl, dH_2_O, ethanol, and acetone, in that order) and dried prior to alpha spectrometry.

*Method 2: TRU-Ag-TEVA separation (final method)*. After MnO_2_ coprecipitation and solubilization, most samples (Table 1) were loaded onto preconditioned TRU cartridges (10 mL, 4 M HCl; Eichrom) to adhere Po, U, and Th (Horwitz et al. 1993). TRU resin (Eichrom) was washed three times (5 mL, 4 M HCl) before eluting Po, U, and Th (10 mL, 0.1 M ammonium bioxalate) into 150-mL glass beakers containing approximately 20 mL of 0.1 M HCl. The eluent was then reduced to prevent interferences from iron (0.5 mL, 20% wt/vol hydroxylamine^·^HCl; 0.1 mL, 1 M ascorbic acid) (Manickam et al. 2010). Samples were incubated (90°C) in a double boiler on a stir plate. A magnetic stir bar and a polished Ag disk (one side coated with acid-resistant acrylic spray-paint) were placed into the beaker. After 2.5 hr, disks were removed and washed (10 mL each 0.1 M HCl, H_2_O, ethanol, and acetone, in that order). The remaining solution was taken to dryness and resuspended (10 mL, 4 M HCl). U and Th were then separated on a TEVA cartridge (Eichrom) using a method developed in our laboratory (Knight et al. 2014). The solution of 4 M HCl containing U and Th was loaded onto a preconditioned TEVA column (10 mL, 4 M HCl). Th does not adhere to the column in these conditions. Therefore, Th was collected in the eluent of the load solution along with an additional column wash (10 mL, 4 M HCl). The column was then washed (25 mL, 4 M HCl) to remove trace Th before U was eluted (5 mL, 0.1 M HCl). Th was precipitated by a rare-earth hydroxide as follows: cerium (Ce; 30 μg), bromocresol purple (1 mL, 0.1%), and H_2_O_2_ (30 μL, 30%). The pH was adjusted to 7 with ammonium hydroxide and left undisturbed (30 min). U sources were prepared by a rare-earth fluoride precipitation by addition of Ce (50 μg), titanium trichloride (1 mL), and hydrofluoric acid (1 mL). U and Th samples were filtered on Eichrom Resolve Filters according to the manufacturer’s recommendation. For a workflow schematic, see Supplemental Material, Figure S1.

**Table 1:** Activity, recovery, and separation method for select radioisotopes analyzed by alpha spectrometry of produced fluids.

Isotope	Activity (mBq/L)	SD of activity (mBq/L)	Recovery (%)	SD of recovery (%)^**^	Days^*a*^	*n*	Method^*b *^
^210^Po	151	3	42	11	21	3	1
	388	12	28	2	50	3	1
	596	10	13	2	70	3	1
	1,000	24	84	6	99	4	2
	4,130	40	77	5	278	4	2
^228^Th	5,750	140	71	2	66	4	2
	6,900	23	87	7	99	4	2
	22,020	850	87	9	278	4	2
^232^U	N/A	N/A	60	3	99	3	2
	N/A	N/A	69	8	278	3	2
^238^U	1.13	0.17	70	3	28	3	3
^235^U	0.14	0.05	70	3	28	3	3
^234^U	2.58	0.88	70	3	28	3	3
Values for activity and recovery represent means. ^***a***^After 0800 hours on 7 May 2013 (CST). ^***b***^Method 1, Sr and Ag autodeposition; method 2, TRU-Ag-TEVA; method 3, TRU-TEVA.							

*Method 3: TRU-TEVA for separation of U and Th*. Reported activities of U in the produced fluids were determined using a method previously developed in our laboratory (Knight et al. 2014). This method differs only slightly from those described above. Briefly, the pellets were dissolved in HNO_3_ (10 mL, 2 M) and TRU resin was preconditioned and washed with HNO_3_ (10 mL, 2 M) in lieu of HCl. This method was investigated but abandoned, as it does not allow for analysis of ^210^Po.

*Isotope dilution alpha spectrometry*. All alpha sources were quantitated by standard isotope dilution techniques and counted in vacuum controlled α-spectrometers [Alpha Analyst (Canberra) or Alpha Ensemble (ORTEC)] as previously described (Knight et al. 2014). Briefly, source-to-detector distances were usually 10 mm, corresponding to a counting efficiency of approximately 18–30%. In some instances, the distance was increased to improve resolution. Radiochemical yields were determined by standard protocols using efficiencies calculated with a NIST traceable, multiline α-spectrometry standard source (E&Z 91005 or Analytics 59956-121). For all samples, thin films were used to prevent daughter recoil contamination of detectors (Inn et al. 2008). Sources were counted for 17–200 hr, as necessary. Standard isotope dilution techniques were used to calculate the activity and recoveries of added controls. In samples where ^232^U and ^230^Th were used, activity of ^228^Th introduced from the ^232^U tracer was subtracted using yield determinations for Th isotopes calculated by ^230^Th. QA/QC included blanks (no added tracers) and laboratory control spikes (LCS).

*Radioactive ingrowth modeling*. Radioactive ingrowth was modeled generally according to the Bateman equation (Bateman 1910) and solved in Microsoft Excel. The derivation and formatting of the Bateman equation was obtained from Choppin et al. (2002).

## Results

**Figure 2:**
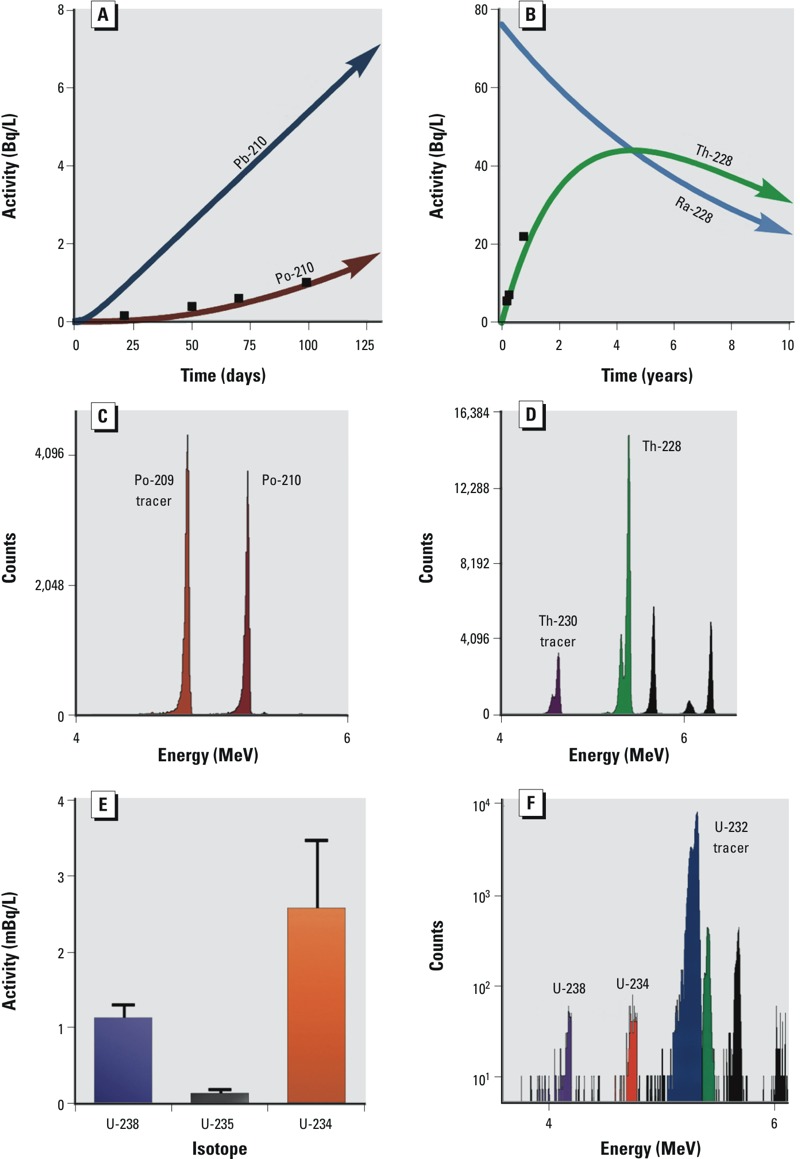
Activity and alpha spectra of Po, Th, and U. (*A*) Theoretical Bateman model of ^210^Pb ingrowth (blue) and ^210^Po (red) given ^226^Ra levels and a system closed to emanation of gaseous radon in produced fluids sample with our empirical data (black squares; error bars subsumed within boxes). (*B*) Theoretical Bateman model of ^228^Th ingrowth (green) and ^228^Ra decay (blue) given ^228^Ra levels and a system closed to emanation of gaseous radon in produced fluids sample with our empirical data in black (error bars subsumed within boxes). (*C*) Representative Po alpha spectrum of ^209^Po tracer (orange) and ^210^Po (red). (*D*) Representative Th alpha spectrum of ^230^Th tracer (purple), ^228^Th (green), and ^228^Th decay products (black). ^232^Th was virtually undetectable by this method. (*E*) Activities of ^238^U (purple), ^235^U (black), and ^234^U (orange) in produced fluids; error bars represent one standard deviation of the determined activity of multiple counts (*n* = 3). (*F*) Representative U alpha spectrum of ^238^U (purple), ^235^U (not labeled), ^234^U (red), ^232^U tracer (blue), and ^232^U tracer decay products (^228^Th green; others black).

**Figure 3:**
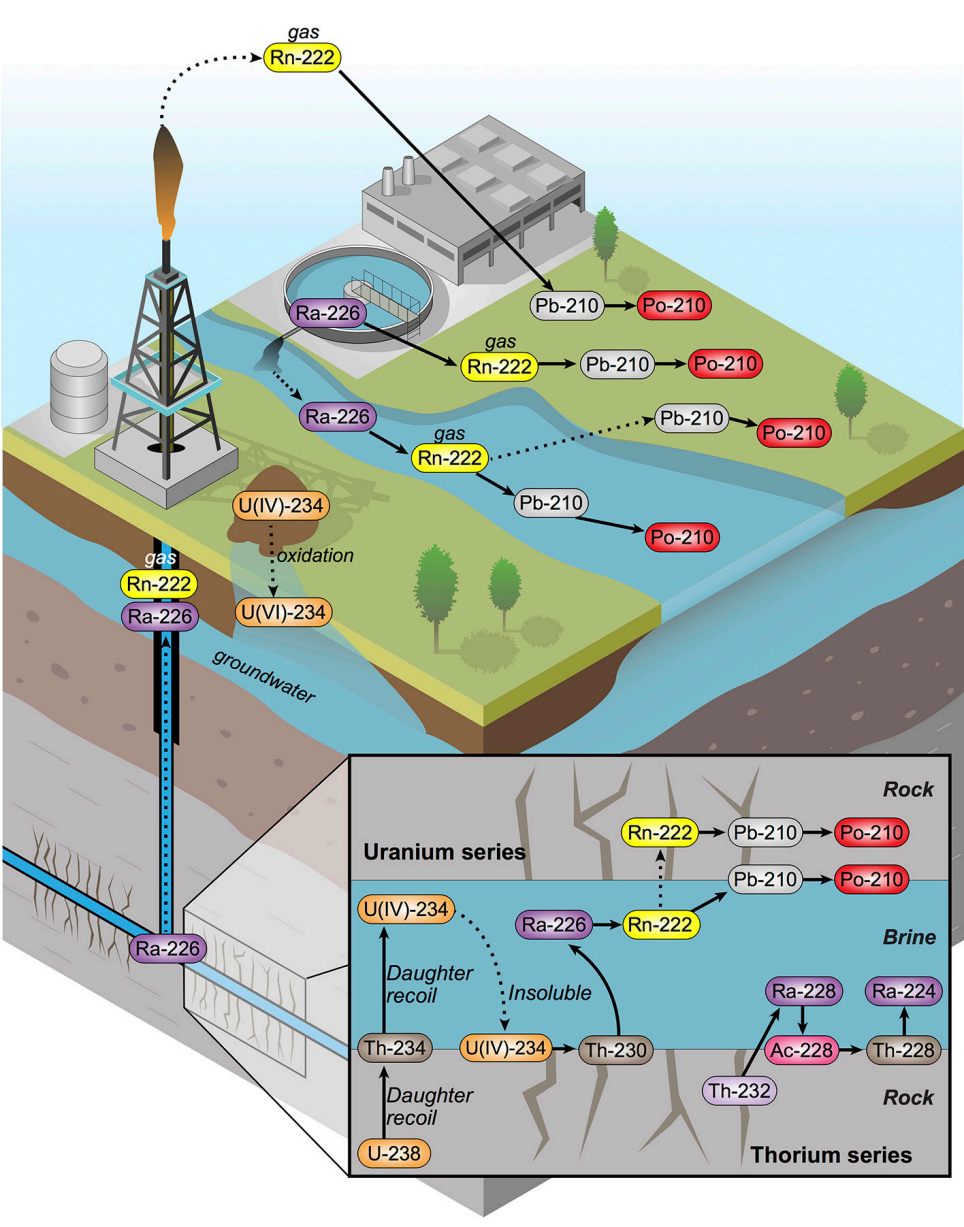
Theoretical model of NORM partitioning and associated waste in Marcellus Shale based on HPGe gamma spectrometry and alpha spectrometry of produced fluids. Solid arrows indicate a radioactive decay or series of radioactive decays. Dashed arrows indicate a physical or chemical partitioning process.

*Radiochemical disequilibria and ingrowth*. Radiochemical yields for the final methodology were Po (81 ± 6%), U (63 ± 8%), and Th (85 ± 9%). The observed concentrations of natural U (^238^U, ^235^U, ^234^U), and Th isotopes (^234^Th, ^232^Th, and ^230^Th) were exceedingly low (< 5 mBq/L). These levels represented < 0.001% of the ^226^Ra radioactivity concentration (670 ± 26 Bq/L; 186 keV peak) in the sample of produced fluids described previously (Nelson et al. 2014). Similarly, we found that the radioactivity concentrations of Ra decay products, including ^228^Th, ^214^Pb, ^214^Bi, ^212^Pb, ^210^Pb, ^210^Po, and ^208^Tl, were initially near detection limits (Figure 2A–D, Tables 1 and 2; see Supplemental Material, “Expanded methods, Polonium-210 ingrowth”). In contrast, subsequent analysis of the same sample of produced fluids over time revealed an increase in the radioactivity concentration of decay products ^210^Po and ^228^Th, which are supported by ^226^Ra and ^228^Ra, respectively (Figure 1; Figure 2A,B). Importantly, the storage drum was hermetically sealed between subsamplings for analysis of radioactive decay products to prevent the release of gaseous radon. Notably, under these conditions, the observed increase in radioactivity concentration of ^210^Po and ^228^Th followed an established radioactive ingrowth model (Bateman equation), which describes the ingrowth of decay products following a separation (radioactive disequilibrium) of decay products from the parent radionuclide at time zero (*t*_0_). From these observations we developed a theoretical model for the geochemical partitioning of NORM in the Marcellus Shale formation, within the context of hydraulic fracturing and associated waste disposal activities (Figure 3). This model serves as a guide for predicting the partitioning and radioactive ingrowth/decay of NORM in the environment surrounding unconventional drilling and hydraulic fracturing operations, as well as in the waste treatment and disposal setting. Importantly, the ultimate fate and transport of NORM in the surface and subsurface environment is site dependent and depends on the potential for release of radon gas; thus, the assessment of the ultimate fate and transport of NORM must be examined on an individual site basis.

**Table 2:** HPGe gamma spectrometry of produced fluids.

Isotope	Activity (Bq/L)	Uncertainty (Bq/L)^*a*^	CL (Bq/L)^*b*^^,^^*c*^	Peaks (keV)
^228^Ac	76	1	0.6	911, 338
^224^Ra	21	3	3	241
^212^Pb	2.4	0.3	0.2	239
^208^Tl	< CL	N/A	1	860
^234m^Pa	< CL	N/A	13	1,001
^234^Th	< CL	N/A	7	63
^226^Ra	670	3	3	186
^214^Pb	256	2	0.8	295, 242, 580, 480
^214^Bi	235	1	0.3	609, 1,120,1,238, 768, 934, 1,385, 1,583, 274
^210^Pb	< CL	N/A	20	46
^40^K	10	1	1	1,461
HPGe analysis was performed using 3 L produced fluids homogenized with BactoAgar in a 3 L Marinelli beaker.^***a***^GammaVision-generated counting uncertainties. ^***b***^At time of acquisition, 1418 hours (CST) on 15 May 2013. ^***c***^Critical level (CL) determined by the Currie limit.				

## Discussion

*Modeling the partitioning NORM in Marcellus Shale*. The partitioning U and Th decay series radionuclides in Marcellus Shale liquid wastes is a function of elemental geochemical behavior—linked with key biogeochemical features of the formation. Like many marine black shale formations, the Marcellus Shale is an ancient seabed that became enriched in U associated with organic matter (Carter et al. 2011; Kargbo et al. 2010; Swanson 1961). Produced fluids from the Marcellus Shale have characteristically high levels of salts, the origin of which has several explanations (Blauch et al. 2009). There are notably low levels of sulfate (SO_4_^2–^) (Osborn and McIntosh 2010), likely due to microbial processes that produce sulfides (S^2–^) (Libes 1992). The ionic strength, reducing environment, and low abundance of SO_4_^2–^ alter the potential for NORM to solubilize in produced fluids. For example, low levels of SO_4_^2–^ and relatively high ionic strength enhance the solubility of Ra, whereas reducing conditions promote precipitation of geochemical species of reduced U, that is, U(IV). Radium decay product radionuclides, such as Pb and Po, are also much more particle reactive and less likely to be extracted through the unconventional drilling and hydraulic fracturing process than decay-series parent Ra isotopes. Thus, differences in the speciation of the elements in the natural decay series govern the likely concentration that will be observed in liquid wastes (as they emerge from depth), following an unconventional drilling and hydraulic fracturing event.

**Figure 4:**
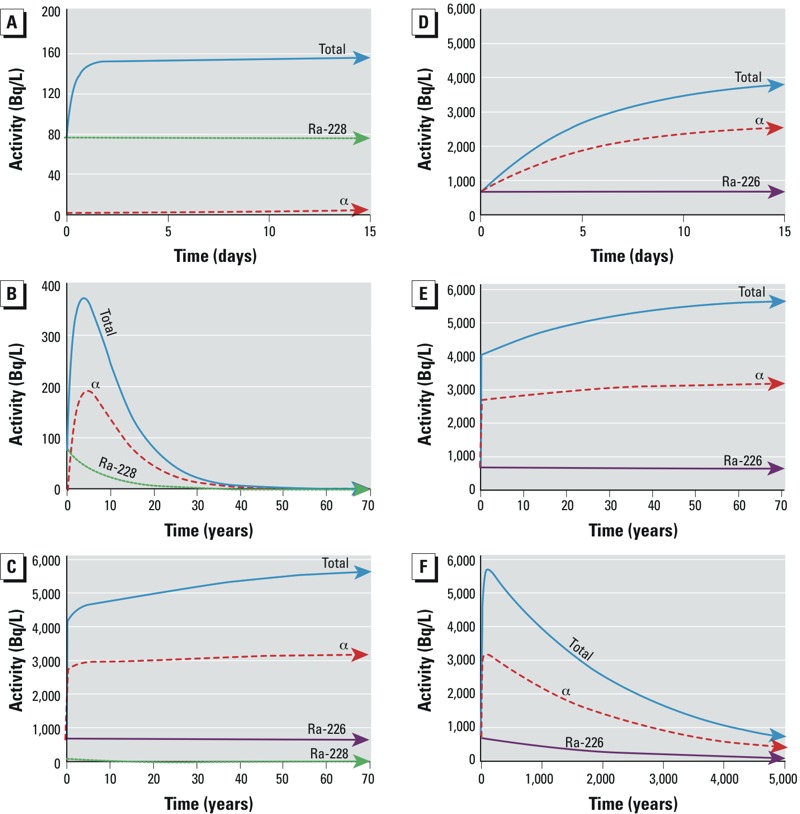
Theoretical Bateman model of Ra decay product ingrowth and decay (system closed to release of gaseous radon) (*A*) 15 days after extraction for ^228^Ra (green dots), associated alpha (red dashes), and total activity (blue); (*B*) 70 years after extraction for ^228^Ra (green dots), associated alpha (red dashes), and total activity (blue); (*C*) 70 years after extraction for ^226^Ra (purple), ^228^Ra (green dots), associated alpha (red dashes), and total activity (blue); (*D*) 15 days after extraction for ^226^Ra (purple), associated alpha (red dashes), and total activity (blue); (*E*) 70 years after extraction for ^226^Ra (purple), associated alpha (red dashes), and total activity (blue); and (*F*) 5,000 years after extraction for ^226^Ra (purple), associated alpha (red dashes), and total activity (blue).

^232^Th Series partitioning. The parent and supporting isotope in the natural Th decay series, ^232^Th (*t*_1/2_ = 1.4 × 10^10^ years), is not expected to undergo oxidation/reduction reactions under natural conditions at depth in the formation, but is nonetheless particle reactive and insoluble in environmental waters and brines (Melson et al. 2012). Accordingly, we observed exceedingly low concentrations of ^232^Th in unfiltered Marcellus Shale produced fluids. However, the decay of ^232^Th produces highly soluble divalent alkaline earth ^228^Ra (*t*_1/2_ = 5.75 years), which has likely been in radioactive secular equilibrium (steady-state) with ^232^Th for many millions of years (Gonneea et al. 2008). As a result, produced fluids are enriched in ^228^Ra (relative to ^232^Th), which is highly soluble in the high-salt-content brines that describe produced fluids. ^228^Ra decays by beta emission to short-lived ^228^Ac (actinium-228; *t*_1/2_ = 6.15 hr), which likely forms insoluble complexes and quickly adsorbs to mineral surfaces at depth—and decays rapidly to form highly insoluble alpha-particle–emitting radionuclide ^228^Th (*t*_1/2_ = 1.91 years) (Hammond et al. 1988). Similar to other Th isotopes, ^228^Th is insoluble in interstitial fluids of shale formations, and its concentration is also low in produced fluids as they emerge from depth. Notably, the large difference in solubility between ^228^Ra and ^228^Th gives rise to a chronometer that has the potential to determine the time when fluids were extracted from the Marcellus Shale (for more information, see Supplemental Material, “Expanded methods, Thorium-228 ingrowth”). As ^228^Th ingrows at a rate related to its half-life, its decay product ^224^Ra (*t*_1/2_ = 3.63 days), rapidly ingrows to steady-state radioactive equilibrium. Rapid ingrowth of ^224^Ra is followed by a series of short-lived radioactive decay products that ultimately decay to stable ^208^Pb (Figure 1). Within this series of relatively short-lived decay products, gaseous ^220^Rn (*t*_1/2_ = 55.6 sec) presents a potential challenge to modeling expected increases in total radioactivity resulting from radioactive ingrowth processes. In contrast, because the half-life of ^220^Rn is so short, migration beyond the immediate vicinity of nuclear formation is likely minimal and disequilibrium is not expected. Thus, in this decay series, the modeled total ^228^Ra-supported radioactivity concentration in produced fluids has the potential to increase to a maximum within 5 years of extraction from the shale formation, followed by a decrease determined by the half-life of ^228^Ra (*t*_1/2_ = 5.75 years) (Figure 4A,B). This suggests that inclusion of the ingrowth and decay of ^228^Ra decay products (particularly ^228^Th) is important for development of appropriate liquid waste management.

^238^U series partitioning. Owing to the geologic history and reducing (anoxic) conditions at depth in the Marcellus Shale formation, parent and supporting radionuclide ^238^U (which, unlike ^232^Th, can be redox active under natural conditions) is likely to be contained in the crystal lattice of minerals or adsorbed to solid phase structures in a reduced highly insoluble (+4) oxidation state (Swanson 1961) (Figures 1 and 3). Thus, geochemical conditions favor adsorption of ^238^U and decay-product actinides (^234^Th, ^234^Pa, and ^234^U) to interstitial surfaces of surrounding minerals (Figures 1 and 3) (Melson et al. 2012), and these radionuclides are likely fixed at depth. In support of these assertions, we observed exceedingly low concentrations of U and Th radionuclides in unfiltered produced fluids from Marcellus Shale (Tables 1 and 2; see also Supplemental Material, “Uranium absent”). Analysis of alpha spectra further revealed an apparent enrichment of ^234^U (relative to ^238^U) in produced fluids, which can likely be explained by alpha-recoil processes (Figure 2E,F) (Fleischer 1980; Osmond et al. 1983). Further investigations of partitioning among relevant phases (filtered/ultrafiltered aqueous, particulate, and solid) will provide more detailed understanding of the speciation of actinides in unconventional drilling liquid wastes.

In contrast to low solubility of ^238^U-series actinides in produced fluids, ^238^U decay product radionuclide ^226^Ra (*t*_1/2_ = 1,600 years) is highly soluble in such fluids. Thus, ^226^Ra becomes enriched in the aqueous phase at depth relative to supporting actinides, with which ^226^Ra has likely been in secular equilibrium (steady state) for many millions of years (Gonneea et al. 2008). Decay product radionuclides of ^226^Ra are concerning because of the long half-life of ^226^Ra, which ensures natural production (via radioactive ingrowth) of decay products for thousands of years (Figure 4C–F). Although ^226^Ra is highly soluble in produced fluids, our observations suggest that ^226^Ra decay product radionuclides (Figures 1 and 3) are relatively insoluble under these conditions and are retained at depth by interactions with mineral phases in the interstitial environment. Although this geochemical behavior results in a very low concentration of ^226^Ra decay products as fluids emerge from depth, the Bateman radioactivity ingrowth equations predict that (in systems closed to the release of gaseous ^222^Rn) the total ^226^Ra-supported radioactivity concentration in produced fluids can increase by a factor > 5 (alpha-particle emissions by a factor of approximately 4) over a period of 15 days following extraction of produced fluids (Figure 4D). Importantly, radioactive ingrowth will continue for decades as longer-lived isotopes (^210^Pb, *t*_1/2_ = 22 years; ^210^Po, *t*_1/2_ = 138 days) approach radioactive equilibrium with ^226^Ra (at a rate related to their own half-lives; Figure 4E). As an example, we compared the Bateman equation–based radioactivity ingrowth model to the observed radioactivity concentration of alpha-emitting radionuclide ^210^Po in sequential analyses of unfiltered, acidified produced fluids from Marcellus Shale that were stored in a hermetically sealed container for several months. The observed increase in radioactivity concentrations of ^210^Po in this sample followed the predicted ingrowth under the conditions described (Figure 2A). Ingrowth of long-lived radioactive ^210^Pb and ^210^Po is important to overall risk assessments in this context because these radionuclides are potentially bioavailable and may accumulate in higher organisms (Bacon et al. 1988; Cherrier et al. 1995; Fisher et al. 1983; Heyraud and Cherry 1983). Thus, the use of ^226^Ra alone to predict total radioactivity concentration in liquid drilling wastes can underestimate the increase in levels that will occur over time and neglects the potential for the bioaccumulation of alpha- and beta-emitting decay product radionuclides in bacteria, plants, and higher organisms.

Similar to the decay product scenario of Th-series Ra isotope ^228^Ra, establishing radioactive equilibrium of decay product radionuclides with parent ^226^Ra is potentially confounded by the presence of a gaseous isotope (i.e., ^222^Rn, *t*_1/2_ = 3.82 days) in the decay series. Further, in this case the half-life of ^222^Rn is sufficiently long to potentially promote migration and separation (disequilibrium) from parent ^226^Ra in systems that are open to the atmosphere (e.g., containment ponds; Figure 3). In these cases, the modeled concentration of ^226^Ra decay products will need to include an assessment of ^222^Rn emanation and decay to accurately portray the total concentration in liquid drilling wastes and the impact of increased ^222^Rn and decay products to surroundings.

## Conclusion

Previous reports that described the radioactivity concentration in flowback, produced fluids, and other materials associated with unconventional drilling and hydraulic fracturing focused on one element—Ra. Our projections suggest that in systems closed to the release of gaseous Rn, estimates based solely on ^226^Ra/^228^Ra will underestimate the total activity present by a factor > 5 within 15 days following extraction as Ra decay product radionuclides ingrow. The level of radioactivity (in a closed ^226^Ra decay product system) will continue to increase and reach a maximum approximately 100 years after extraction (Figure 4F). At this time, when the long-lived ^210^Pb and its decay products have reached equilibrium with ^226^Ra, the total radioactivity will have increased by a factor > 8. Although this projection assumes that losses of Rn and other geochemically derived disequilibria are negligible, the physical process of ingrowth begins again at any time of Ra separation (e.g., sulfate treatment at wastewater treatment plants), and the total activity unavoidably increases as decay product radionuclides ingrow. Thus, long-lived, environmentally persistent Ra decay products (^228^Th, ^210^Pb, ^210^Po) should be considered carefully as government regulators and waste handlers assess the potential for radioactive contamination and exposures.

NORM is emerging as a contaminant of concern in hydraulic fracturing/unconventional drilling wastes, yet the extent of the hazard is currently unknown. Sound waste management strategies for both solid and liquid hydraulic fracturing and unconventional drilling waste should take into account the dynamic nature of radioactive materials. Methods designed to remove Ra from hydraulic fracturing waste may not remove Ra decay products because these elements (Ac, Th, Pb, Bi, Po isotopes) have fundamentally different physicochemical properties (Kondash et al. 2014; Zhang et al. 2014). Future studies and risk assessments should include Ra decay products in assessing the potential for environmental contamination in recreational, agricultural, and residential areas, as well as in developing a more detailed understanding of the accumulation of these radionuclides in higher organisms.

## Supplemental Material

(1 MB) PDF
