# Supplementary material for: Understanding the Radioactive Ingrowth and Decay of Naturally Occurring Radioactive Materials in the Environment: An Analysis of Produced Fluids from the Marcellus Shale
Source: Environ Health Perspect. 2015 Apr 2;123(7):689–96. doi: 10.1289/ehp.1408855 (PMC4492269; doi:10.1289/ehp.1408855)
Supplement: (1 MB) PDF [file ehp.1408855.s001.acco.pdf]

Environ Health Perspect
doi: 10.1289/ehp.1408855

Note to Readers: ***EHP*** **strives to ensure that all journal content is accessible to all readers. However, some figures and Supplemental Material published in** ***EHP*** **articles may not conform to 508 standards due to the complexity of the information being presented. If you need assistance accessing journal content, please contact ehp508@niehs.nih.gov. Our staff will work with you to assess and meet your accessibility needs within 3 working days.**

Supplemental Material

Understanding the Radioactive Ingrowth and Decay of Naturally Occurring Radioactive Materials in the Environment: An Analysis of Produced Fluids from the Marcellus Shale

Andrew W. Nelson, Eric S. Eitrheim, Andrew W. Knight, Dustin May, Marinea A. Mehrhoff, Robert Shannon, Robert Litman, William C. Burnett, Tori Z. Forbes, and Michael K. Schultz

Table of Contents

Figure S1. Schematic of rapid separation of U, Th and Po.

Figure S2. Representative alpha spectra. Panels **(A-D)** are representative spectra collected by *Method 1: TRU-Ag-TEVA*: **(A)** polonium fraction without ^{209}Po tracer, **(B)** polonium fraction with ^{209}Po tracer, **(C)** thorium fraction without ^{230}Th tracer, **(D)** thorium fraction with ^{230}Th tracer. Panels **(E-F)** are representative spectra collected by *Method 2: TRU-TEVA*: **(E)** uranium fraction with ^{232}U tracer linear scale, **(F)** uranium fraction with ^{232}U tracer log scale.

Expanded methods

Polonium-210 ingrowth

Thorium-228 ingrowth

Uranium absent

References

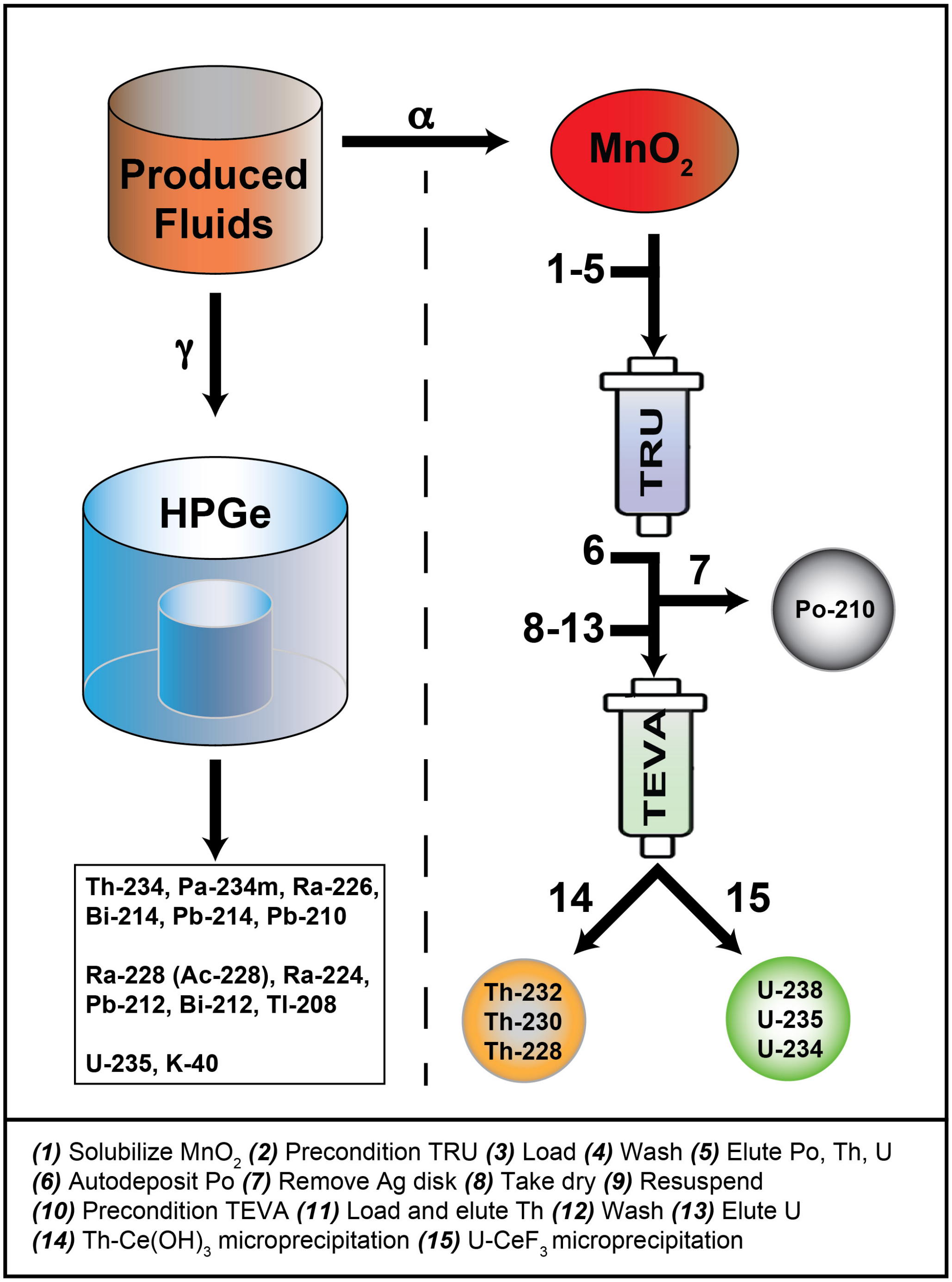

Figure S1. Schematic of rapid separation of U, Th and Po.

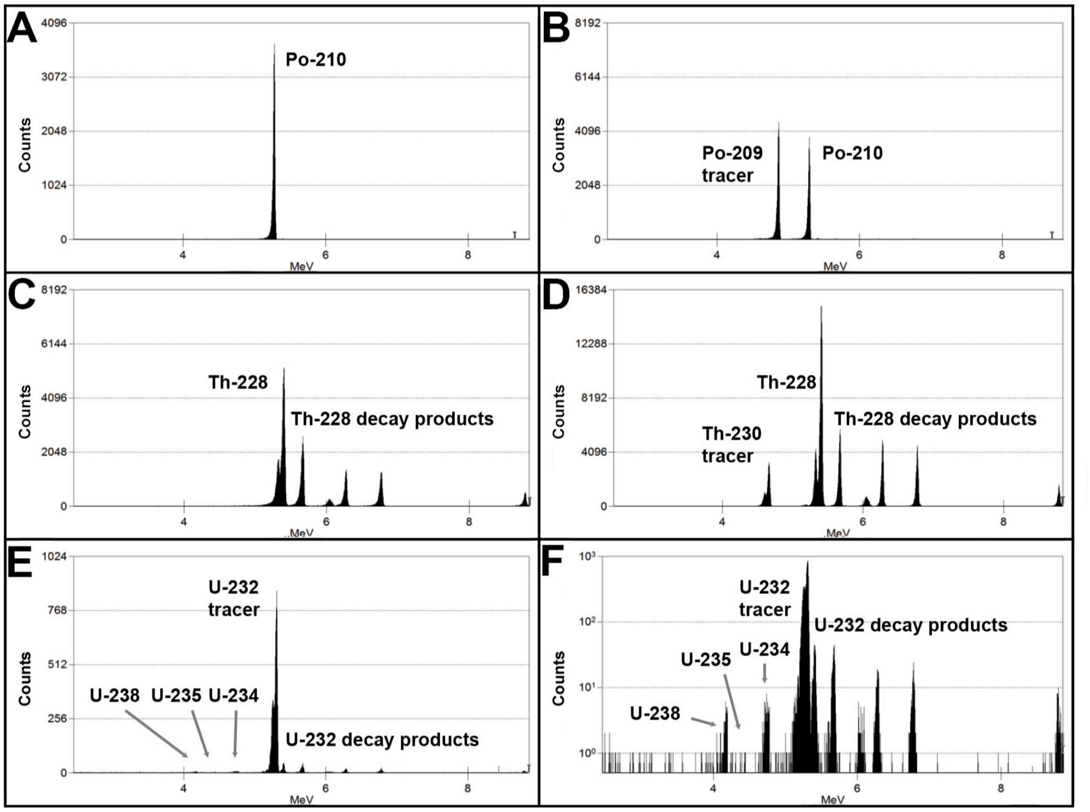

Figure S2. Representative alpha spectra. Panels **(A-D)** are representative spectra collected by *Method 1: TRU-Ag-TEVA*: **(A)** polonium fraction without ^{209}Po tracer, **(B)** polonium fraction with ^{209}Po tracer, **(C)** thorium fraction without ^{230}Th tracer, **(D)** thorium fraction with ^{230}Th tracer. Panels **(E-F)** are representative spectra collected by *Method 2: TRU-TEVA*: **(E)** uranium fraction with ^{232}U tracer linear scale, **(F)** uranium fraction with ^{232}U tracer log scale.

Expanded methods

Polonium-210 ingrowth

The long-lived ^{238}U ($t_{1/2}$ = 4.5 x 10^9 years) in the formation supports the activity of ^{226}Ra, which then supports ^{210}Pb ($t_{1/2}$ = 22.3 years) and ^{210}Po. Thus, these elements should be present (and of equal activity) in the Marcellus Shale. Given that ^{226}Ra was 670 Bq/L, we expected similar activities of ^{226}Ra decay products. Yet, when we directly measured ^{210}Pb by gamma spectrometry, its activity was below the critical level (Currie Limit, 14 Bq/L). We acknowledge this critical level may be unacceptably high for many applications. The high critical level is due to several reasons, first, the relatively high activity of Ra isotopes and decay products create a large Compton scatter, which buries the low energy peak of ^{210}Pb (46 keV, 4%). Emission data for ^{210}Pb were extracted from the NuDat 2 Database [National Nuclear Data Center (NNDC) 2013]. Secondly, the high levels of ions in produced fluids attenuate gamma emissions thereby further reducing counting efficiency of the low intensity peak (Landsberger et al. 2013). Importantly, ^{210}Pb was *not in secular equilibrium* with its parent, ^{226}Ra, which led us to investigate the levels of ^{210}Po (the final radioactive species in the ^{238}U decay series). Similarly, experiments indicated ^{210}Po was *not in secular equilibrium* with either ^{226}Ra or ^{210}Pb. When we measured ^{210}Po levels ~2 months later, we noticed levels had increased approximately 450%. This ingrowth, follows the theoretical Bateman equation with the assumption that all decay products of ^{226}Ra are initially absent (Figure 2A).

Thorium-228 ingrowth

Initial experiments indicated that levels of thorium isotopes (^{232}Th, ^{230}Th, ^{228}Th) were negligible. Yet, over time the levels of ^{228}Th steadily increased. In this sample, ^{228}Th is supported by ^{228}Ra and its *ingrowth* can be modeled on a *transient equilibrium* model (Figure 2B). Given that Th is

generally insoluble in environmental waters (Kumar et al. 2013) and in produced fluids Ra is soluble, the transient equilibrium model of ^{228}Ra/^{228}Th has the potential serve as a 'forensic' tool to determine when samples removed from the Marcellus Shale (for up to ~ 10 years (Schmidt and Cochran 2010)). The ^{228}Ra/^{228}Th system provides key advantages over other tools including (1) the chemical disequilibrium introduced by the poor solubility of Th (2) the relative ease of measuring ^{228}Th and ^{228}Ra (via ^{228}Ac) and (3) the relatively short half-live of ^{228}Th ($t_{1/2}$ = 1.9 years) that allows for a chronometer that may be used within a matter of weeks.

Uranium absent

In addition to Ra decay products, we investigated levels of ^{238}U, ^{235}U, and ^{234}U in the produced fluids. Although U is often analyzed by mass spectrometry, the method we developed provides the advantage of simultaneous determinations of multiple U, Po, and Th isotopes. Given the high level of ^{226}Ra (^{238}U decay product), we were surprised to find levels of U isotopes less than 5 mBq/L (n=4), which is nearly 5-log lower than the activity of ^{226}Ra (Figure 2). There are very few peer-reviewed reports of U activities in produced fluids; however, the notably lower levels of U compared to ^{226}Ra is similar to data from the PA Department of Environmental Protection (Barbot et al. 2013). Our analysis indicates there is a slight enrichment of ^{234}U compared to ^{238}U (^{234}U/^{238}U = 2.3), which is common in groundwater and indicative of daughter recoil (Osmond *et al.* 1983).

References

Barbot E, Vidic NS, Gregory KB, Vidic RD. 2013. Spatial and temporal correlation of water quality parameters of produced waters from Devonian-age shale following hydraulic fracturing. Environ Sci Technol 47:2562-2569.

Kumar A, Singhal R, Rout S, Narayanan U, Karpe R, Ravi P. 2013. Adsorption and kinetic behavior of uranium and thorium in seawater-sediment system. J Radioanal Nucl Chem 295:649-656.

Landsberger S, Brabec C, Canion B, Hashem J, Lu C, Millsap D, et al. 2013. Determination of ^{226}Ra, ^{228}Ra and ^{210}Pb in NORM products from oil and gas exploration: Problems in activity underestimation due to the presence of metals and self-absorption of photons. J Environ Radioact 125:23-26.

NNDC (National Nuclear Data Center). 2013. NuDat 2 database. Available: http://www.nndc.bnl.gov/nudat2/ [accessed 17 February 2015].

Osmond J, Cowart J, Ivanovich M. 1983. Uranium isotopic disequilibrium in ground water as an indicator of anomalies. Intl J Appl Radiat and Isot 34:283-308.

Schmidt S, Cochran J. 2010. Radium and radium-daughter nuclides in carbonates: a brief overview of strategies for determining chronologies. J Environ Radioact 101:530-537.
